# Biological Synthesis of Silver Nanoparticles Using Lactobacillus Probiotic Bacterium and Evaluation of Their Cytotoxicity Against Oral Squamous Cell Carcinoma Cell Line: AgNPs Cytotoxicity Against Squamous Cell Carcinoma

**DOI:** 10.31661/gmj.v12i.2905

**Published:** 2023-12-17

**Authors:** Mohadeese Pourhaji, Farid Abbasi, Aliyeh Sehatpour, Ronak Bakhtiari

**Affiliations:** ^1^ Department of Oral Medicine, School of Dentistry, Shahed University, Tehran, Iran; ^2^ School of Public Health, Tehran University of Medical Sciences, Tehran, Iran

**Keywords:** Biosynthesis, Silver Nanoparticles, quamous Cell Carcinoma, Cytotoxicity, Anticancer, Lactobacillus Acidophilus

## Abstract

**Background:**

The applications of nanotechnology have greatly increased in the recent years. Nanotechnology can be used for diagnosis and treatment of many conditions in medicine and dentistry. The aim of this paper is assessment the cytotoxicity of silver nanoparticles (AGNPs) synthesized employing Lactobacillus acidophilus against human oral squamous cell carcinoma (OSCC) cell line.

**Materials and Methods:**

In this in vitro, experimental study, AgNPs were biologically synthesized by using L. acidophilus, and characterized by dynamic light scattering (DLS), scanning electron microscopy (SEM), transmission electron microscopy (TEM), ultraviolet-visible (UV-V) spectroscopy and Fourier-transform infrared (FTIR) spectroscopy. The methyl thiazolyl tetrazolium (MTT) test was performed to assess the cytotoxic effects of AgNPs in 3.125, 6.25, 12.5, 25, 50, and 100 μg/mL concentrations within 24 hours.

**Results:**

Synthesis of AgNPs was confirmed by visual perception of dark brown color variation (from achromatic) and maximum UV-V absorption at 428 nm. TEM and SEM indicated the spherical form of AgNPs with a median size of 397 nm. FTIR spectroscopy showed the presence of functional groups from the cells involved in the reduction process. The MTT assay indicated that the biosynthesized nanoparticles made a decrease of cell livability in a concentration dependent method.

**Conclusion:**

AgNPs produced by Lactobacillus acidophilus have the potential to inhibit OSCC cell line.

## Introduction

Oral cancer refers to malignancies of the lips, oral cavity, and the upper respiratory system [1]. More than 90% of the malignancies of the oral cavity are related to oral squamous cell carcinoma (OSCC) [2]. Radical surgery is the treatment of choice for SCC. However, adjuvant radiotherapy and chemotherapy may be beneficial in some cases, depending on the location and stage of lesio [3]. Radical surgery can cause significant morbidity particularly in the head and neck region, and compromise esthetics and function. The other commonly used modalities for treatment of head and neck cancers, such as chemotherapy and radiotherapy, are often ineffective and even harmful due to lack of specificity, severe side effects, and emergence of clinical drug resistance [4]. Despite the great advances made in diagnosis and treatment of various cancer types, the 5-year survival rate of OSCC remains at 50% and it is still one of the common causes of death worldwide [5]. Therefore, there is an increasing demand for more effective methods to more efficiently combat OSCC [6].

Use of nanoparticles (NPs) is one of the most recent methods for cancer treatment. Nanotechnology is a field of research and innovation that involves synthesis of materials and devices on the scale of atoms and molecules and with a size ranging from 10 to 100 nm. The physical and chemical characteristics of materials can substantially differ as they transform into NPs [7]. Silver nanoparticles (AgNPs) are one of the most common metallic nanomaterials applied in medicine due to their unique characteristics [8]. The anticancer properties of AgNPs and their potential to inhibit the cancer cells have been proven in various studies [9]. For instance, a study showed that biosynthesized AgNPs were efficient anticancer agents that induced apoptosis of colon cancer cells (HCT-116) [10].AgNPs can be synthesized by several methods. Chemical and physical methods are less favorable due to the use of toxic chemical agents or high pressure in their procedures. The biological synthesis of AgNPs is often preferred because it is cost-effective, commercially viable, and environmentally clean [11]. In many studies, AgNPs were synthesized by using plants, fungi, yeasts, and bacteria [12][13]. L. acidophilus is one of the most common types of probiotic microorganisms that can be found in dairy products such as milk, yogurt, and cheese. L. acidophilus also exists in some parts of the human body such as the intestines and the oral cavity, and plays a vital role in health of human [14]. In the current investigation, AgNPs were biosynthesized by employing L. acidophilus, and their cytotoxicity was assessed against OSCC cell line.

## Materials and Methods

### 1. Biosynthesis of AgNPs using L.Acidophilus

Lyophilized Lactobacillus acidophilus (ATCC 4356) strain was purchased from the Persian Type Culture Collection of Iran and cultured in 50 mL of sterile de Man, Sharpe and Rogosa broth. The composition of this broth was as follows: 10 g beef extract, 10 g proteose peptone, 20 g dextrose, 5 g yeast extract, 2 g ammonium citrate, 1 g polysorbate 80, 5 g sodium acetate, 2 g dipotassium phosphate, 0.05 g manganese sulfate and 0.1 g magnesium sulfate. The culture was incubated at 37°C in anaerobic conditions for 24 hours using an anaerobic jar. After incubation, the microbial suspension was harvested by centrifugation (5000 rpm for 10 minute). The collected cell supernatant was then filtered applying Whatman grade 40 filter paper and transferred into sterile tubes. AgNPs were biosynthesized regarding to the scheme explained by Thomas et al., [15] with some modifications. Approximately 2 mL of the suspension was mixed with 0.09 g of AgNO3 and 20 mL of distilled water. The suspension was then incubated at 37°C for 24 h with agitation at 150 rpm in the dark. The confirmation of AgNPs synthesis was performed by the colorimetric assay (color change of the solution).

### 2. Biosynthesized AgNPs Characterization

#### 2.1. Spectral Analysis of Ultraviolet-visible (UV-V)

Synthesis of AgNPs was confirmed by UV-V spectroscopy. For this purpose, 300 µL of the AgNP suspension was transferred into a cuvette, and the cuvette was placed in a spectrophotometer for testing. The absorption was read by the UV-V spectrophotometer (Agilent, spectrophotometer, USA) at 200 to 700 nm wavelength range.

### 2.2. Scanning Electron Microscopy (SEM)

The morphology and size of synthesized AgNPs were observed by SEM. AgNPs were dissolved in water and the obtained suspension was placed on the gold grid. The samples were gold sputter-coated (sputter coater SBC-12, KYKY, China) and the coated surfaces were examined under a SEM microscope (XL30; Philips, Japan) operating at 30 kV with 10-5 Torr pressure. The micrographs were acquired from the samples.

### 2.3. Transmission Electron Microscopic (TEM) Observation

The morphology and size of synthesized NPs were confirmed by TEM observation, by a TEM microscope (LEO 906; Zeiss, Germany) with an accelerating voltage of 120 kV. A drop of AgNP suspension was fixed on the carbon coated copper grids and dried at temperature of room. The particle size distribution of AgNPs was assessed by employing Image J software version 1.8.0 (https://imagej.nih.gov/ij/download.html).

### 2.4. Fourier-transform Infrared (FTIR) Spectroscopy

The functional groups types of AgNPs were defined employing Spectrum Two FT-IR (PerkinElmer, Germany). The potassium bromide (in 1:100 ratio) was used to dilute the AgNP powder and the FTIR spectroscope was operated in diffuse reflectance mode. The spectra were scanned in the range of 400 to 4500 cm-1 at 4 cm-1 resolution; then, the functional groups were identified according to the references.

### 2.5. Dynamic Light Scattering (DLS)

The size and distribution width of particles were measured by DLS (Day Petronic Biological Company, Tehran, Iran). Zeta potential analyzer and SZ-100z Dynamic light scattering was used. It was made by Horiba Jobin Jyovin company in Japan. The size distribution of nanoparticles was measured with the INSO16247 standard. Synthesized AgNPs were used as a solution in water dispersant and placed in the cell of the device, and then the analysis was performed on the desired sample.

### 2.6. Cell Culture

OSCC cell line (HSc-4) was purchased from the Pasteur Institute cell bank, Tehran, Iran. RPMI 1640 was used as the basic medium to culture the tumor cells; 100 mL of 10% fetal bovine serum, 1 mL antibiotic solution (100 µg/mL streptomycin and 100 U/mL penicillin), 1 L deionized water, and 3.7 g sodium bicarbonate were added to 13.4 g of RPMI 1640 medium.

The cells were stored in a humidified incubator with 5% CO2 and 37C temperature.

### 2.7. Cell Viability Assay

The Trypan blue staining of HSC-4 cells was performed. The cells were counted and the percentage of viability was calculated. Next, the cells were seeded in a 96-well-plate (100 µL culture medium containing 10000 cells in each well). Next, the cells were incubated at 37℃ in a 5% humidified CO2 incubator for one day. At high concentration Ag is toxic for human beings; nonetheless in low concentration it is nontoxic. El-Nagar et al. [16] and Baetke et al. [17] stated that AgNPs toxicity depended on concentration, size, and surface composition. Therefore, the concentration of AgNPs was chosen based on the previous researches [8][12][10][18][19][20][21][22]. Silver nanoparticles were prepared at 100, 50, 25, 12.5, 6.25 and 3.125 μg/mL concentrations. Next, the cultured cells were treated with the prepared AgNPs concentrations and incubated for one day. The methyl thiazolyl tetrazolium (MTT) solution was ready by dissolving 50 mg of 3-(4,5- Dimethylthiazol-2-yl)-2,5-diphenyltetrazolium bromide (MTT powder) in 10 mL of phosphate-buffered saline. Next, 100 µL of the prepared solution was appended to each well and incubated for 3 hours. Next, 100 µL of dimethyl sulfoxide was joined to each well to solve the crystals of formed formazan. After 30 minutes at room temperature, absorption was read at 570 nm wavelength by an ELISA reader (Bio Tek). The percentage of viability of OSCC cells was achieved by the following formulas:


\textit{Cytotoxicity percentage} = \left( 1 - \frac{\textit{mean absorbance of toxicant treated cells}}{\textit{mean absorbance of negative control}} \right) \times 100

\textit{Viability percentage} = 100 - \textit{cytotoxicity percentage}

### 3. Statistical Analysis

Statistical analysis was done by SPSS version 16 [23]. The normality of data was checked by Kolmogorove-Smirnow test. The one-way ANOVA was used to analyze of the data, and the results were declared as average ± standard deviation. Statistically significant was considered for P-value<0.05.

## Results

**Figure-1:**
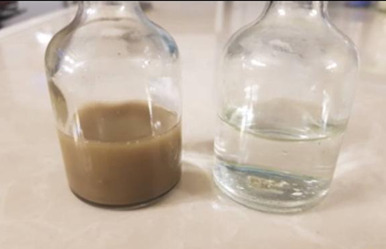
Figure 1. Biosynthesis of silver nanoparticles (AgNps) using bacterial supernatant.

**Figure-2:**
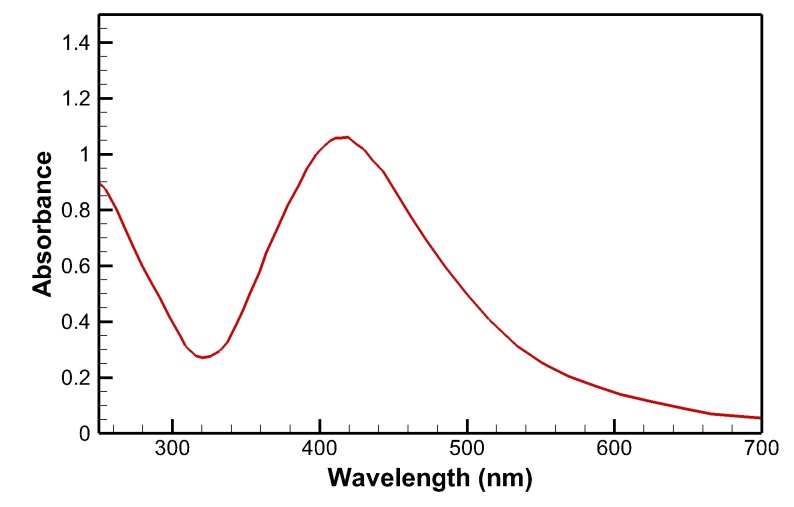
Figure 2. UV-V spectroscopy of biosynthesized silver nanoparticles(AgNPs)

**Figure-3:**
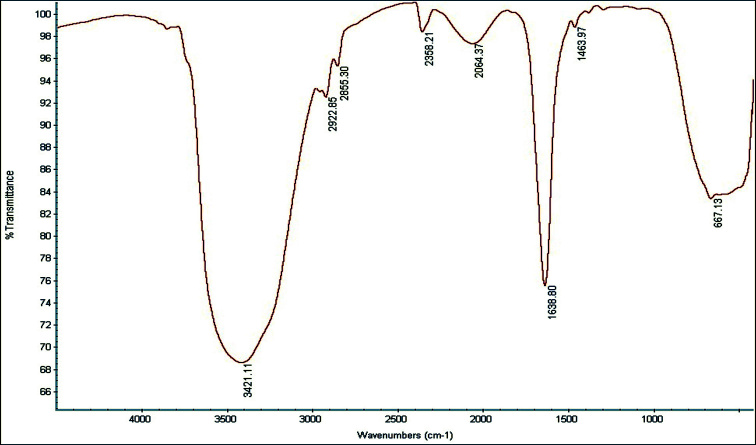
Figure 3. FTIR analysis of biosynthesized silver nanoparticles (AgNPs)

### Characterization of Biosynthesized AgNPs

The dark brown color change (from achromatic) indicated successful biosynthesis of AgNPs (Figure-1). The dark brown color change (from achromatic) indicated successful biosynthesis of AgNPs (Figure-1). Furthermore, presence of an absorption peak at 428 nm in UV-V spectroscopy confirmed the synthesis of AgNPs through the reduction reaction (Figure-2). The images obtained by TEM and SEM observations showed that the biosynthesized AgNPs were in the shape of sphere (Figure-3and4) with a mean size of 397 nm (Figure-5). The functional groups of AgNPs were detected by the FTIR spectra. The peaks at 3421 cm-1 and 667 cm-1 may be due to the O-H stretching vibration. In addition, a peak at 1638 cm-1 corresponded to C=O in the amide functional group (Figure-6). DLS results showed that the average size of AgNPs is 397 nm.

### Cell Viability Assay Results

The cytotoxic influence of biosynthesized AgNPs on OSCC cell line was evaluated employing the MTT assay. The results indicated that AgNPs decreased cell viability after 24 hours and in a dose-depended manner. The 3.125, 6.25, 12.5, 25, 50 and 100 µl/mL concentrations decreased the cell viability by 85.34%±0.15% (P-value<0.05),74.26%±0.33%, 41.63%±0.61%,31.75%±0.67%, 23.12%±0.95% and 12.83%±0.65% (P-value<0.001), successively (Figure-7).

## Discussion

**Figure-4:**
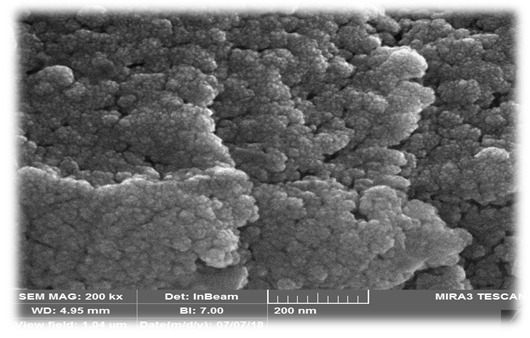
Figure 4. SEM micrograph of biosynthesized silver nanoparticles (AgNPs)

**Figure-5:**
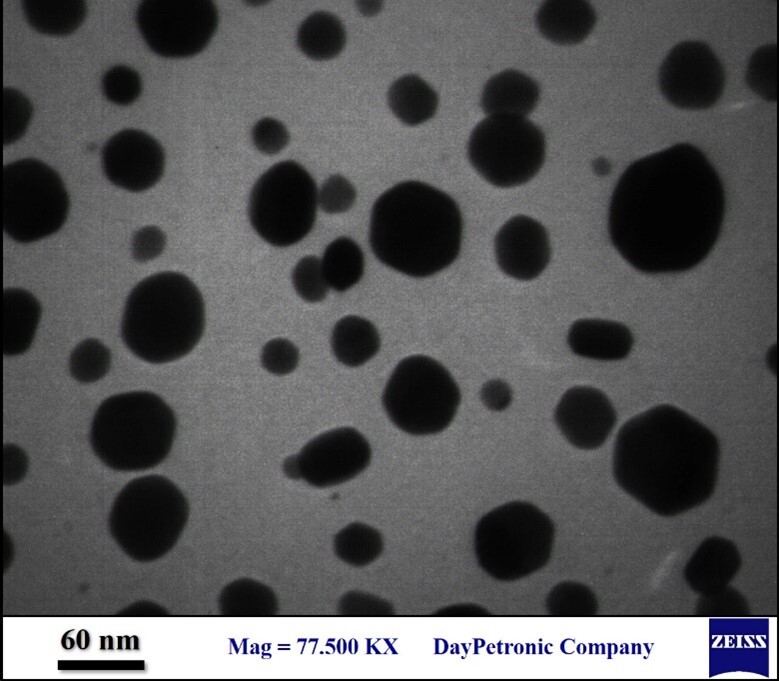
Figure 5. TEM micrograph of biosynthesized silver nanoparticles (AgNPs)

**Figure-6:**
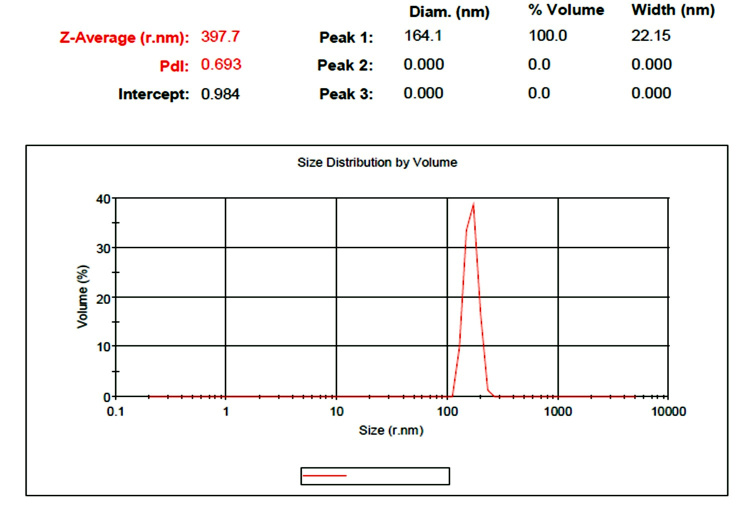
Figure 6. DLS analysis of biosynthesized silver nanoparticles (AgNPs).

**Figure-7:**
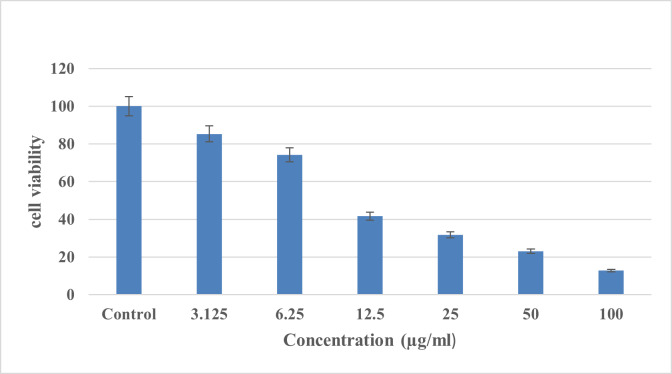
Figure 7. Cytotoxicity of silver nanoparticles )AgNPs( against oral squamous cell carcinoma cell line. Outcomes are stated as average ± standard deviation as compared with the group of control (***: P-value<0.001, **: P-value<0.01, *: P-value<0.05, n=3).

Biodegradability, decreased drug side effects and biocompatibility are the advantages of NPs [24][25]. NPs allow for protection from quick metabolism and clearance controlled drug release. NPs are also help to improved patient compliance because of a reduced rate of drug administration in comparison with unencapsulated drugs [24]. However, possible carrier toxicity is a disadvantage for NPs [26]. Preparation of NPs on a big industrial scale with reproducible properties is one of their limitation. The stability of NPs is another limitation [27][28]. However, the NPs can simultaneously deliver agents with various physical-chemical properties and mitigate adverse influences. A proper NPs-based supply system should to satisfy these requests: (a) co-load various molecules in adequate amounts; (b) overcome biologic fences without dropping its bioactivity; (c) distribute goods at the purpose time and site; (d) have the capability to aim specific cell type or tumor; (e) display additive or synergistic properties; and (f) should apply safe, economic and efficient preparation methods [24]. In the current investigation, AgNPs were biosynthesized applying L. acidophilus, and their cytotoxic effect on OSCC cell line was evaluated. Metallic NPs such as gold, silver, selenium, palladium, and platinum are extensively used in medicine and dentistry and have many applications as in diagnostic imaging procedures, targeted drug delivery systems, tissue engineering, antibacterial activity, wound healing, gene therapy, and cancer treatment [29][30]. AgNPs are more commonly used than other metallic NPs because of their favorable characteristics such as electrical conductivity, chemical stability, low sintering temperature, low cost, and optimal antibacterial, antifungal, antiviral, and antioxidant effects [31][32]. Jia et al., [33] used triethanolamine as a chemical reducing agent to produce AgNPs 40 nm in size. Several methods can be used to synthesize AgNPs including biological, chemical and physical methods.

However, biosynthesis of AgNPs is environmentally and economically friendly, and is a more convenient alternative to chemical and physical approaches [33][34]. Li et al., [11] successfully synthesized AgNPs using the Capsicum annuum plant. In the current study, the AgNPs synthesis was affirmed by observing a dark brown color alteration and presence of a peak at 428 nm wavelength in UV-V spectroscopy. According to TEM and SEM observations, and the results of DLS, the biosynthesized AgNPs in our study were in the shape of sphere with a mean size of 397 nm. The biological synthesis of AgNPs has been an interesting research topic. For instance, in 2015, Mata et al. synthesized AgNPs using the Abutilon Indicum plant which was similar to our study. AgNPs Synthesis was affirmed by a color alteration from light green to yellowish-brown.

UV-V analysis also showed a peak at 455 nm. The data obtained from SEM, TEM, and DLS indicated that NPs were in the shape of sphere and their size was in the range of 1-300 nm [35]. In another work by Hamida et al, AgNPs were synthesized by an innovative Cyanobacteria Desertifilum sp. The AgNPs synthesis in this study was affirmed by observing a change of color from pale yellow to dark brown; also the plasmon resonance peak of the synthesized NPs surface was at 421 nm. Under TEM and SEM, AgNPs were in the shape of sphere with a range of diameter from 4.5 to 26 nm [18]. Different sizes and shapes of AgNPs can be synthesized using various species of bacteria due to their different reducing enzymes. Some other factors can also modulate the shape and size of AgNPs such as the concentration of biomolecules, temperature, pH, and reduction time, among others [36]. This can explain the size difference of AgNPs in our study and other similar studies.

In the current study, the functional groups of AgNPs were detected using FTIR spectroscopy. The FTIR spectra had peaks at 3421 cm-1 and 667 cm-1 that represented the O-H stretching vibration of polysaccharides, and a peak at 1638 cm-1 which was related to C=O in the amide functional group. The present results were highly similar to those of Khandel et al, who synthesized AgNPs by using a fungus [21]. In the current investigation, the MTT assay was used to evaluate the cytotoxicity of AgNPs against OSCC cell line. This method is far superior to the dye exclusion technique such as the Trypan blue dye exclusion assay, since it is safe, easy to perform, and has a high reproducibility [37]. Devi and Bhimba [38] used the MTT assay to evaluate the anticancer activity of AgNPs against Hep-2, MF7, HT20, and Vero cell line. The outcomes displayed that the maximum concentration of AgNPs used in this study which was 250 µl/mL decreased the viability of Hep-2, MCF, HT29, and Vero cell line by 11.47%, 12.78%, 12.45% and 34.18%, respectively. Sulaiman et al, in another study, indicated that treatment of HL-60 cell line with 2 mmol/L of biosynthesized AgNPs increased the number of dead cells to 85% after 24 hours of incubation, which was similar to our result [39]. Information obtained from a study done by Inbathmizh et al. indicated that 1000 µg/mL of biosynthesized AgNPs decreased the viability of HEP G2 cells by 16.39% [22].

Generally, the results of the above-mentioned studies were in line with our study. However, a lower concentration of AgNPs synthesized in the current study indicated higher cytotoxicity compared with above similar studies [22][39]. Yakop et al., [40] assessed the cytotoxicity of AgNP-C. nutans with concentrations ranging from 0.75 to 3 µg/mL, against OSCC cell line, which is the same cell type we used in the current investigation. The MTT assay outcomes exposed that the IC50 concentration of AgNPs was 1.61. The cytotoxicity of AgNPs against the cells may vary depending on the differences in size and concentration of nanoparticles, duration of exposure of cells, and type of cells used in the procedure [20]. Rudrappa et al., [41] successfully displayed the potential cytotoxic nature of P-AgNPs against GBM U118 MG cell line by causing a rise in late and early apoptosis cells population. Yakop et al., [40] also showed the apoptotic effects of nanoparticles on HSC-4 cell line by a rise in Bax/Bcl-2 protein ratio. In the current study, the apoptotic effects of biosynthesized AgNPs on the cancer cell line were not evaluated; which calls for future studies on this topic.

According to the outcomes of several investigations carried out in this field, NPs can have significant cytotoxic effects against cancer cells; although further research is required to fully describe the mechanisms involved in anticancer and antimicrobial activities and the toxicity of these particles.

The NPs have been applied successfully in small-scale research laboratories. However, the huge scale industrial of NPs has not been prosperous for numerous reasons. The industrial manufacturing of NPs faces various limits include which affects the chemical, physical, batch-to-batch variability, and product performance qualities. Moreover, it is a time-consuming and hard process that contains numerous processes. Extra stages of difficulty are typically associated to the extra testing performed previous to, during, and next the manufacturing, storing, and clinical uses as well as the lack of well controlled production practices [42][43]. Newly, the production process of drug-loaded NPs has been revolutionized by the most advanced microfluidic systems [42]. The employing of NPs to expand drug delivery has significantly impacted several biome dical regions. It has been displayed that the NPs improve biodistribution and stability of therapeutic agents, overcome limits to cellular uptake and tissue in objective sites in vivo, and decrease systemic toxicity related to non-encapsulated agents. In spite of the extensive preclinical study on NPs, their conversion to the clinical has progressed slowly. Future investigations should emphasis on these limitations. This needs continuous collaborations and communications among experts in all steps of pharmaceutical progress, containing preclinical and clinical applications as well as toxicological assessments [24].

## Conclusion

The biosynthesized AgNPs showed significant cytotoxic effects on OSCC cell line after 24 hours. However, extra in vivo investigations are needed to confirm the anticancer properties of AgNPs and their successful and safe application in the clinical settings. Investigation of the effects of other NPs (i.e., Mg and Al) or their combinations on OSCC cell line together with in vivo study will be an interesting topic for the researchers.

## Conflict of Interest

The authors declare that they have no known competing financial or personal relationships that could have appeared to influence the work reported in this paper.
